# Supplementary material for: Assessing the viability of transplanted gut microbiota by sequential tagging with D-amino acid-based metabolic probes
Source: Nat Commun. 2019 Mar 21;10:1317. doi: 10.1038/s41467-019-09267-x (PMC6428874; doi:10.1038/s41467-019-09267-x)
Supplement: Supplementary file 1 — Supplementary Information [file 41467_2019_9267_MOESM1_ESM.pdf]

Supplementary Information

Assessing the Viability of Transplanted Gut Microbiotas by Sequential Tagging with D-Amino acid-based Metabolic Probes

(Wang et al.)

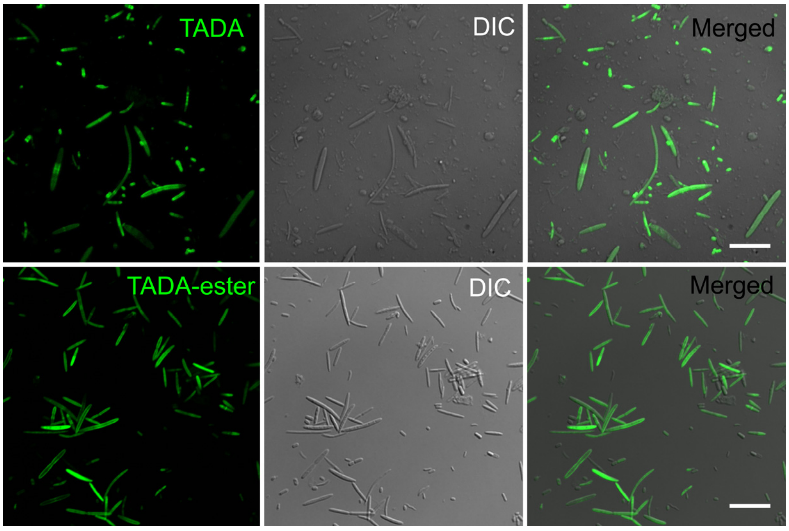

Supplementary Figure 1. Confocal fluorescence microscopy showed that mouse gut microbiotas could be strongly labeled by both TADA and TADA-ester *in vivo*. DIC, differential interference contrast. Scale bars, 10 μm.

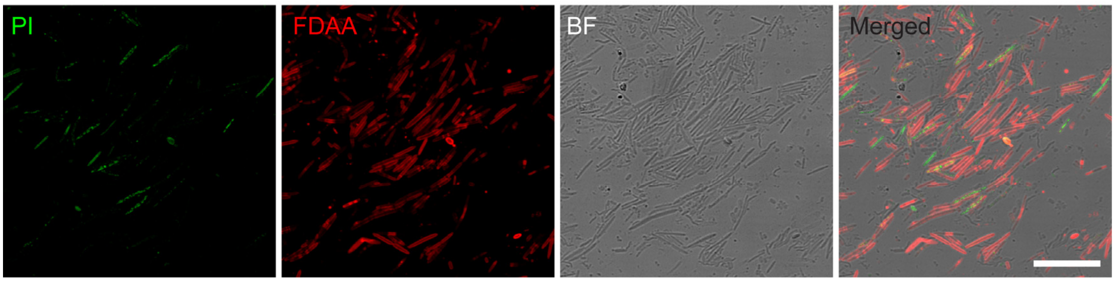

Supplementary Figure 2. Confocal fluorescence microscopy revealed many of the bacteria not labeled by FDAA (Cy5ADA-amide, red) were dead bacteria, which were stained by propidium iodide (PI, green). Representative images from at least three independent experiment are shown. Scale bar, 20 μm.

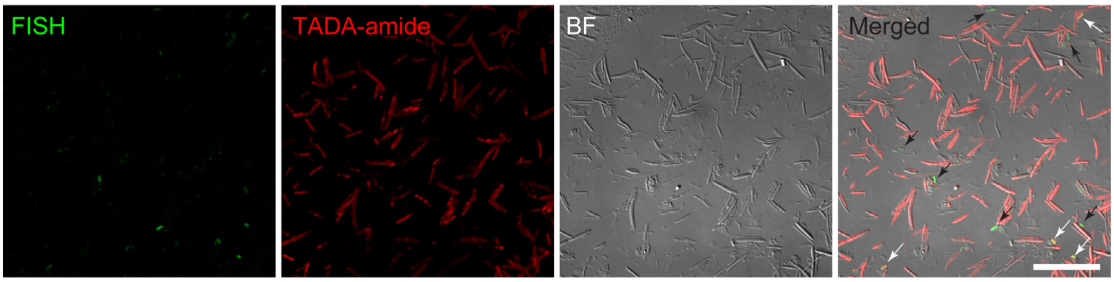

Supplementary Figure 3. Confocal fluorescence microscopy analysis of the TADA-amide labeled microbiota stained by a *Bacteroidetes*-targeting FISH probe. The FDAA labeled mouse microbiota (red) was stained with a *Bacteroidetes*-targeting FISH probe (green). Some doubly labeled *Bacteroidetes* were observed (white arrow), suggesting some bacteria in the *Bacteroidetes* phylum could be labeled by FDAA. However, some of the bacteria not labeled by TADA-amide but by FISH (black arrow) were seen, proving that some bacterial species in the *Bacteroidetes* phylum were not stainable by TADA-amide *in vivo*. BF, bright field. Representative data from at least three independent experiment are shown. Scale bar, 20 μm.

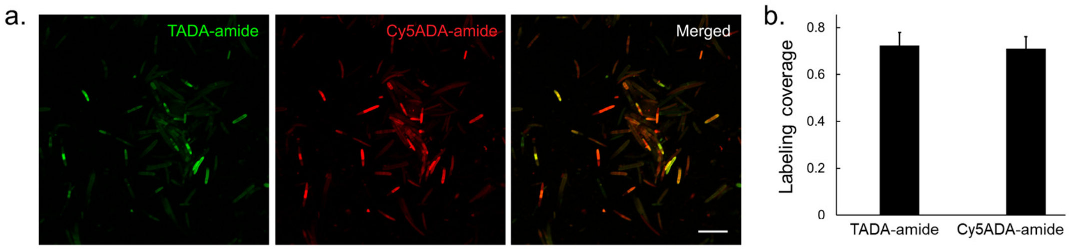

Supplementary Figure 4. a). Confocal fluorescence microscopy analysis of the mouse cecal microbiota labeled simultaneously by two FDAAs. Two gavages with an interval of 3 h were applied to C57BL/6 mice containing TADA-amide and Cy5ADA-amide with concentrations of 0.5 mM each. The cecum microbiotas were collected, washed and then analyzed. b). Statistical analysis of the labeling coverage for gut microbes labeled with the two FDAAs revealed by flow cytometry. Representative data from at least three independent experiment are shown. Scale bar, 10 μm. Mean ± s.d. are presented for n=3.

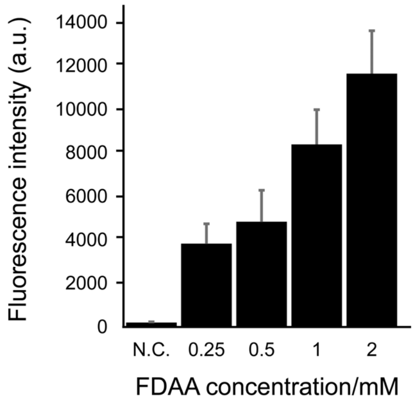

Supplementary Figure 5. FDAA labeling of the mouse gut microbiotas was dose-dependent. Two gavages with an interval of 3 h were applied to C57BL/6 mice (three in each group) containing indicated concentrations of TADA-amide. The cecum microbiotas were collected and the fluorescence intensities of the labeled microbiotas ($\lambda ex/\lambda em$ = 535/565 nm, slit width=10 nm) were measured on a microplate reader (BioTek Synergy Mx, Winooski, VT, U.S.). Mean ± s.d. are presented for n=3.

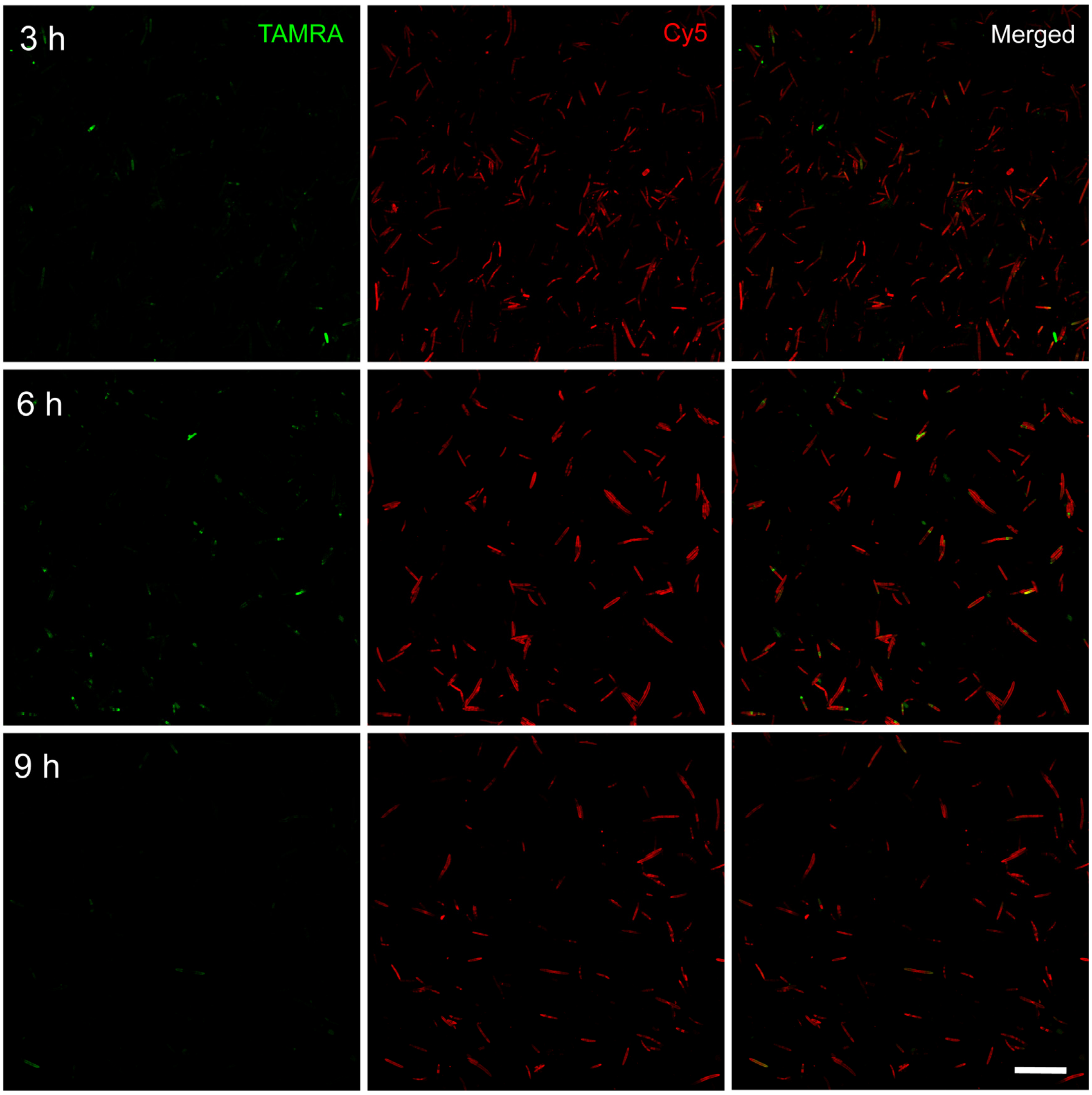

Supplementary Figure 6. Confocal fluorescence microscopy showed that the donor microbiota's fluorescence decreased quickly 3-9 h after the transplantation, followed by another 6 h for the Cy5ADA-amide labeling. Representative images from at least three independent experiment are shown. Scale bar, 20 µm.

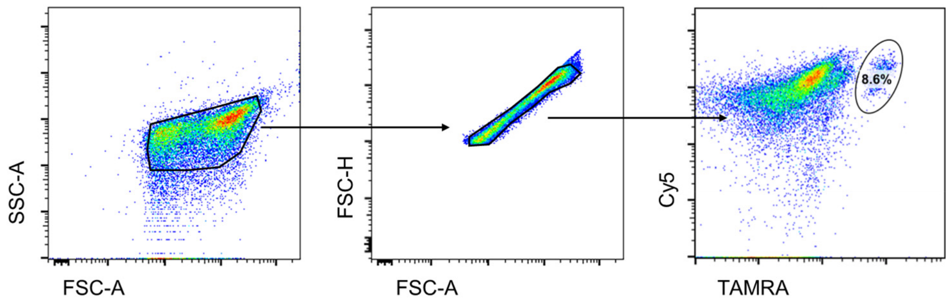

Supplementary Figure 7. Gating strategies used for sorting two-colored bacterial populations (Figure 3b).

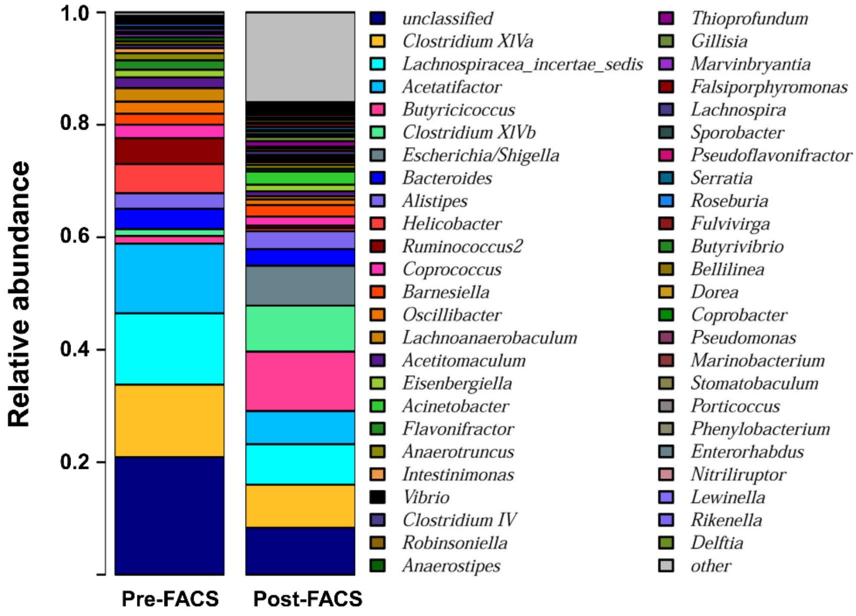

Supplementary Figure 8. 16S rDNA sequencing of the bacteria before and after bacterial sorting revealed that several bacterial genera were enriched in the transplantation survivors.

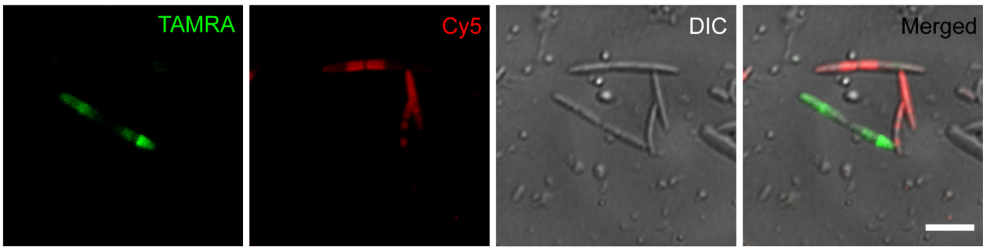

Supplementary Figure 9. Confocal fluorescence microscopy revealed the transplanted bacteria that did not survive (green) and the original bacteria in the recipient's gut (red). Scale bar, 5 µm.

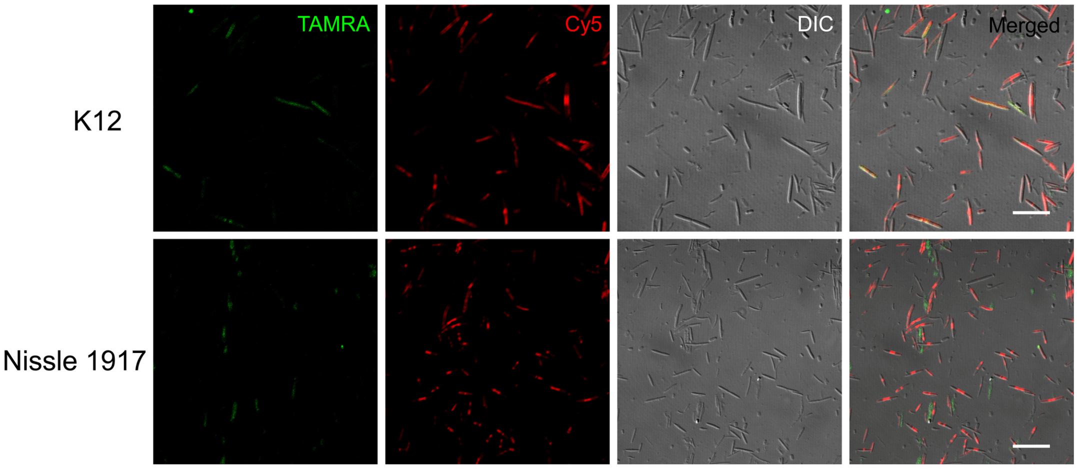

Supplementary Figure 10. Confocal fluorescence microscopy showed that different *E. coli* strains survived differently in transplantation. Following transplantation into mouse gut, more two-colored bacteria were observed in the K12-gavaged microbiota than in Nissle 1917-gavaged, suggesting that K12 had better viability than Nissle 1917 during transplantation. Scale bars, 10 µm.

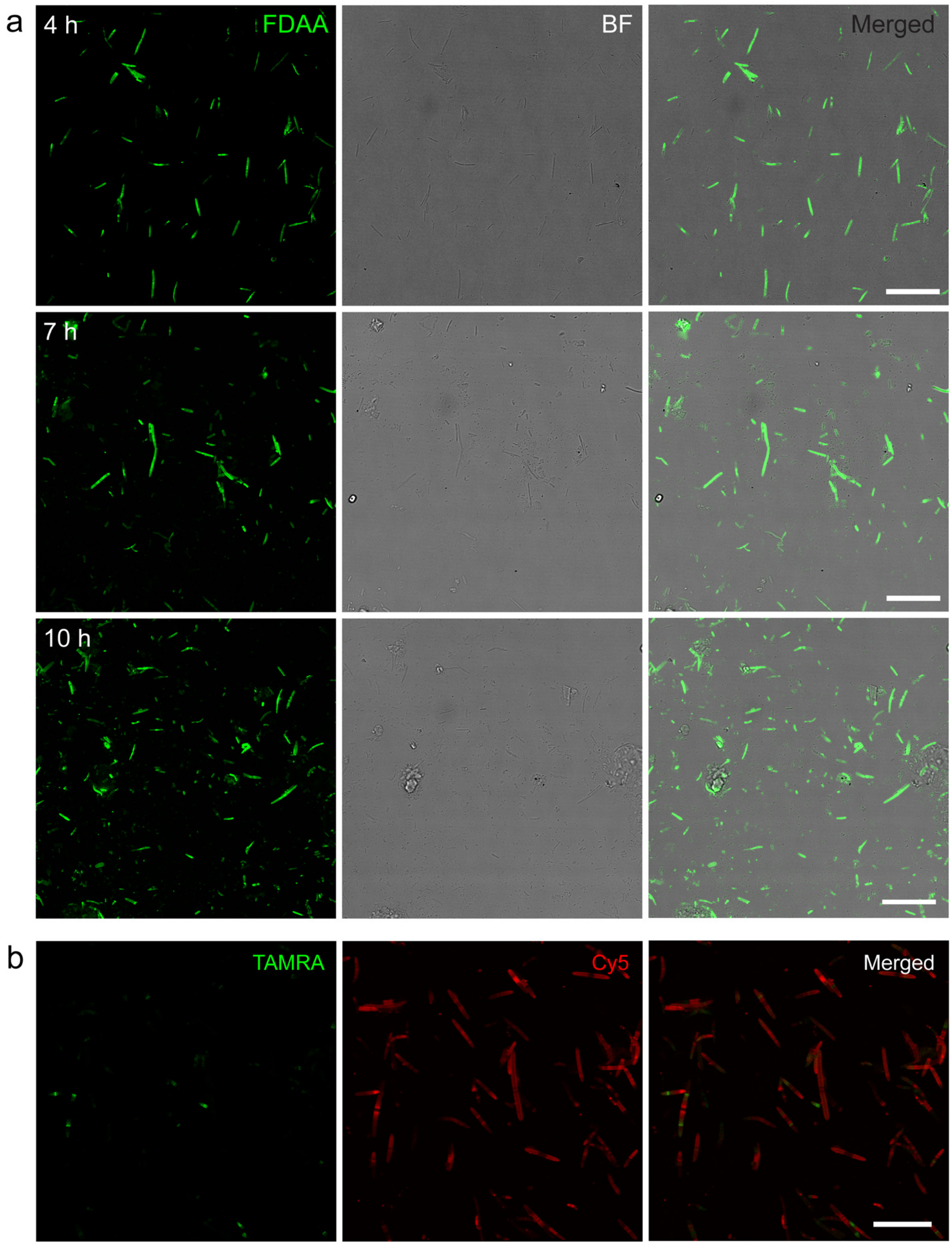

Supplementary Figure 11. STAMP labeling and analysis of the mouse fecal microbiota transplantation. Confocal fluorescence microscopy showed that the bacteria in mouse fecal microbiota showed strong labeling from 4-10 h after the first FDAA (TADA-amide) gavage (a), and their viability in FMT could be assessed by our STAMP strategy (b). Representative images from at least three independent experiment are shown. Scale bars, 20 µm.

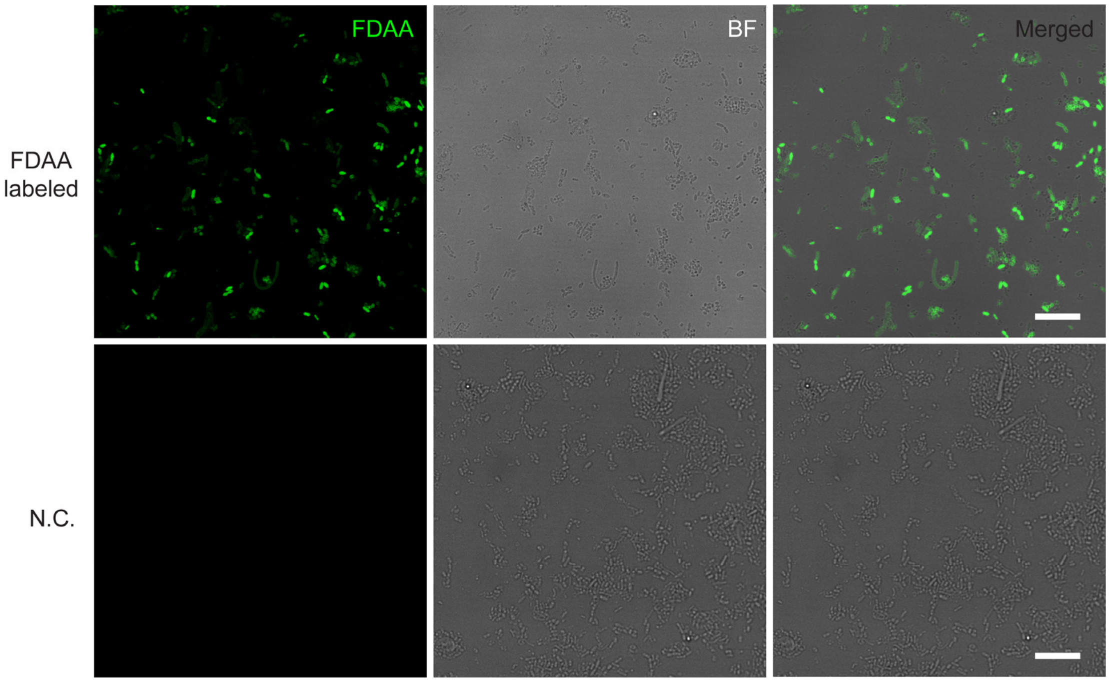

Supplementary Figure 12. Confocal fluorescence microscopy analysis of the human fecal microbiota labeled *in vitro* by FDAA (TADA-amide). Representative data from at least three independent experiment are shown. Scale bars, 10 µm.

Supplementary Table 1. The primers used in this study.

Primer name	Sequence
338F	5'- ACTCCTACGGGAGGCAGCAG-3'
806R	5' -GGACTACHVGGGTWTCTAAT-3'
