# Supplementary material for: Assessing the viability of transplanted gut microbiota by sequential tagging with D-amino acid-based metabolic probes
Source: Nat Commun. 2019 Mar 21;10:1317. doi: 10.1038/s41467-019-09267-x (PMC6428874; doi:10.1038/s41467-019-09267-x)
Supplement: Supplementary file 3 — Reporting Summary [file 41467_2019_9267_MOESM3_ESM.pdf]

nature research

Corresponding author(s): Xing Chen
Chaoyong James Yang

Reporting Summary

Nature Research wishes to improve the reproducibility of the work that we publish. This form provides structure for consistency and transparency in reporting. For further information on Nature Research policies, see Authors & Referees and the Editorial Policy Checklist.

Statistical parameters

When statistical analyses are reported, confirm that the following items are present in the relevant location (e.g. figure legend, table legend, main text, or Methods section).

n/a	Confirmed	
☒	☐	The exact sample size (n) for each experimental group/condition, given as a discrete number and unit of measurement
☐	☒	An indication of whether measurements were taken from distinct samples or whether the same sample was measured repeatedly
☒	☐	The statistical test(s) used AND whether they are one- or two-sided<br>*Only common tests should be described solely by name; describe more complex techniques in the Methods section.*
☒	☐	A description of all covariates tested
☒	☐	A description of any assumptions or corrections, such as tests of normality and adjustment for multiple comparisons
☒	☐	A full description of the statistics including central tendency (e.g. means) or other basic estimates (e.g. regression coefficient) AND variation (e.g. standard deviation) or associated estimates of uncertainty (e.g. confidence intervals)
☒	☐	For null hypothesis testing, the test statistic (e.g. F, t, r) with confidence intervals, effect sizes, degrees of freedom and P value noted<br>*Give P values as exact values whenever suitable.*
☒	☐	For Bayesian analysis, information on the choice of priors and Markov chain Monte Carlo settings
☒	☐	For hierarchical and complex designs, identification of the appropriate level for tests and full reporting of outcomes
☒	☐	Estimates of effect sizes (e.g. Cohen's d, Pearson's r), indicating how they were calculated
☐	☒	Clearly defined error bars<br>*State explicitly what error bars represent (e.g. SD, SE, CI)*

Our web collection on statistics for biologists may be useful.

Software and code

Policy information about availability of computer code

Data collection	CytExpert software (version 2.0) and FACSDiva (version 8.0.1) were used in flow cytometry data collection.
Data analysis	FlowJo software (V 10.0.8r1) was used for flow cytometry data analysis. Excel and Prism were used for data plotting.

For manuscripts utilizing custom algorithms or software that are central to the research but not yet described in published literature, software must be made available to editors/reviewers upon request. We strongly encourage code deposition in a community repository (e.g. GitHub). See the Nature Research guidelines for submitting code & software for further information.

Data

Policy information about availability of data

All manuscripts must include a data availability statement. This statement should provide the following information, where applicable:

- Accession codes, unique identifiers, or web links for publicly available datasets
- A list of figures that have associated raw data
- A description of any restrictions on data availability

The data that support the findings of this study are available from the corresponding author upon reasonable request.

Field-specific reporting

Please select the best fit for your research. If you are not sure, read the appropriate sections before making your selection.

☒ Life sciences ☐ Behavioural & social sciences ☐ Ecological, evolutionary & environmental sciences

For a reference copy of the document with all sections, see nature.com/authors/policies/ReportingSummary-flat.pdf

Life sciences study design

All studies must disclose on these points even when the disclosure is negative.

Sample size	Sample sizes were determined based upon accepted conventions within the field.
Data exclusions	No data was excluded from analysis.
Replication	All experiments were independently repeated and all attempts to replicate the experiments were successful.
Randomization	Mice were allocated into experimental groups randomly.
Blinding	Investigators were blinded to group allocation during data collection and analysis.

Reporting for specific materials, systems and methods

Materials & experimental systems

n/a	Involved in the study	
☒	☐	Unique biological materials
☒	☐	Antibodies
☒	☐	Eukaryotic cell lines
☒	☐	Palaeontology
☐	☒	Animals and other organisms
☐	☒	Human research participants

Methods

n/a	Involved in the study	
☒	☐	ChIP-seq
☐	☒	Flow cytometry
☒	☐	MRI-based neuroimaging

Animals and other organisms

Policy information about studies involving animals; ARRIVE guidelines recommended for reporting animal research

Laboratory animals	Male C57BL/6 mice at the age of 6-8 weeks were used.
Wild animals	The study did not involve wild animals.
Field-collected samples	The study did not involve samples collected from the fields.

Human research participants

Policy information about studies involving human research participants

Population characteristics	Healthy volunteer, no antibiotics administered in the past three months.
Recruitment	The donor of fecal microbiota was a healthy male volunteer at the age of 30 years.

Flow Cytometry

Plots

Confirm that:

- [x] The axis labels state the marker and fluorochrome used (e.g. CD4-FITC).
- [x] The axis scales are clearly visible. Include numbers along axes only for bottom left plot of group (a 'group' is an analysis of identical markers).
- [x] All plots are contour plots with outliers or pseudocolor plots.
- [x] A numerical value for number of cells or percentage (with statistics) is provided.

Methodology

Sample preparation	Mouse ceca were dissected with a pair of 4.5-inch iris scissors in 1ml of degassed phosphate buffer saline (dPBS). The minced tissues and digesta were filtered with cell strainers to remove most of the non-bacterial debris. The filtrate was then centrifuged and the bacterial pellets were then washed with 2 ×1.5 ml dPBS by centrifugation (10,000 × g, 2 min), and resuspended in dPBS for flow cytometry analysis.
Instrument	Flow cytometry was performed on CytoFLex (Beckman Coulter Life Sciences, Indianapolis, IN, U.S.) and Aria II flow cytometer (BD Biosciences, San Jose, CA, U.S.).
Software	CytExpert software (version 2.0) and FACSDiva (version 8.0.1) were used in flow cytometry data collection. FlowJo software (V 10.0.8r1) was used for data analyses.
Cell population abundance	The relative abundance of sorted cell populations was pointed out in the figure containing FACS data. Purity of the isolated bacteria was not confirmed due to the quenching of the samples' fluorescence signals during sorting.
Gating strategy	Labeled bacteria were identified with flow cytometry plots of logFSC versus logSSC and then gated on fluorescence

- [x] Tick this box to confirm that a figure exemplifying the gating strategy is provided in the Supplementary Information.
